# Comparative Flammability and SEM–EDS Characterization of Contemporary, Aged and Charred Oak Wood from Historic Timber Structures

**DOI:** 10.3390/ma19163365

**Published:** 2026-08-07

**Authors:** Andrzej Jurecki, Wojciech Grześkowiak, Beata Klimek, Marek Wieruszewski

**Affiliations:** 1 Faculty of Forestry and Wood Technology, Poznań University of Life Sciences, Wojska Polskiego 28, 60-637 Poznań, Poland; andrzej.jurecki@up.poznan.pl (A.J.); wojciech.grzeskowiak@up.poznan.pl (W.G.); 2 Faculty of Civil Engineering and Architecture, Lublin University of Technology, 20-618 Lublin, Poland; b.klimek@pollub.pl

**Keywords:** historic timber, oak wood, *Quercus robur*, fire behaviour, mass loss calorimeter, heat release rate, SEM–EDS, charred wood, conservation diagnostics

## Abstract

Historic timber combines structural function with heritage value, yet its fire behaviour is inferred from contemporary wood data. This study provides an integrated, condition-based assessment of contemporary oak (*Quercus robur* L.) and historic oak from a nineteenth-century half-timbered building in Swołowo, Poland. Mass loss calorimetry (MLC) at 35 and 50 kW/m^2^ was combined with scanning electron microscopy (SEM) and SEM coupled with energy-dispersive X-ray spectroscopy (SEM–EDS). The aim was to determine whether surface preservation state explains differences between ignition sensitivity and cumulative heat release. Historic oak ignited earlier than contemporary oak, especially when the tested surface was degraded; at 50 kW/m^2^, time to ignition decreased from 25.33 s for contemporary oak to 18 s for non-degraded historic oak and 5.33 s for degraded historic oak. However, total heat release was lower for historic variants and final mass loss remained comparable. SEM showed roughness, fibrillation, delamination, fractured cell-wall edges and secondary porosity in aged and charred zones. SEM–EDS indicated C- and O-dominated preserved areas, whereas degraded and charred surfaces were enriched in inorganic elements. The results show that ignition propensity and total fire contribution should be evaluated separately, and that condition rather than age alone is critical for historic oak assessment.

## 1. Introduction

Wooden elements in historic buildings have a dual role: they remain structural components while also documenting historic construction technology, workmanship and material authenticity. This dual status makes the assessment of historic timber more complex than the assessment of new construction wood, particularly when fire-safety interventions may conceal, alter or replace original fabric [1,2,3,4]. In timber-framed and half-timbered buildings, visible oak members are often central to the heritage value of the structure; therefore, decisions concerning exposure, protection or replacement should be based on material evidence rather than on age alone.

Historic wood refers to wood originating from existing or dismantled historical buildings, engineering structures, furnishings, or works of art that possess documented historical, architectural, or cultural significance. Unlike archaeological wood, which has been buried or submerged for extended periods and undergoes extensive physicochemical degradation under anoxic conditions, historical wood has generally remained in service under atmospheric conditions. Consequently, it retains much of its original anatomical structure, although its physical, mechanical, and chemical properties may be altered by long-term natural ageing, repeated environmental exposure, biological deterioration, and previous conservation treatments. The extent of these changes depends on the wood species, age, service conditions, and maintenance history. Owing to its cultural heritage value and unique material characteristics, historical wood requires dedicated research to evaluate its current properties, conservation status, and potential for restoration or reuse while preserving its historical authenticity. Historic timber is not a homogeneous material category. Individual members may differ in species, density, growth characteristics, surface machining, previous loading, moisture history, biological deterioration, salt contamination, smoke exposure and repair history. Natural ageing and long-term service can modify wood through weathering, leaching, photodegradation, cracking, insect activity and mineral deposition [5,6,7,8,9,10]. These processes do not necessarily make historic timber uniformly more combustible; rather, they create local surface conditions that may influence ignition, charring and heat release under external heat flux.

During fire exposure, wood undergoes drying, pyrolysis, ignition and char formation. The char layer can slow heat transfer to the underlying material, but its protective efficiency depends on continuity, mechanical stability and surface morphology [11,12,13,14]. A rough, cracked or fibrillated historic surface may have a larger active area and may include thin cell-wall fragments that heat more rapidly than compact tissue. Such features may reduce time to ignition even when total heat release remains comparable to or lower than that of contemporary wood.

The present study combines the flammability data of contemporary and historic oak wood with SEM-based microstructural mapping and SEM–EDS elemental interpretation. The aim is to determine whether the surface and near-surface condition of aged and charred oak explains the observed fire-test behaviour, especially the distinction between early ignition sensitivity and overall heat contribution. The study was designed as a condition-based interpretation relevant to conservation diagnostics and fire-safety assessment of historic timber structures [15,16,17].

SEM was selected to characterize the surface and near-surface microstructure because ignition is initiated at the exposed surface and may be affected by vessel geometry, cell-wall continuity, fibrillation, cracking, delamination and secondary voids. SEM–EDS was used as a complementary local screening method to determine whether morphologically altered zones also exhibited elemental heterogeneity or inorganic enrichment. Similar microscopy-based approaches have been applied to degraded and archaeological wood when destructive sampling is restricted [18,19,20,21,22,23,24]. The two methods provide complementary evidence: SEM documents morphology, whereas SEM–EDS links local elemental signals to visible particles, deposits, cracks and altered wall fragments. SEM–EDS does not identify crystalline phases or individual salts; therefore, the elemental maps were interpreted as field-specific evidence of chemical heterogeneity rather than direct phase identification.

A key limitation of many assessments of historic timber is the implicit assumption that chronological age is itself a sufficient proxy for fire performance. This assumption is methodologically weak because ageing processes are heterogeneous, surface-selective and strongly dependent on service environment. In the present study, the working hypothesis was therefore that the early ignition response of historic oak is controlled primarily by the preservation state of the exposed surface and near-surface zone, whereas cumulative heat release is additionally governed by density, available combustible mass, moisture content, heat transfer and char formation.

The specific objectives were (i) to compare MLC-derived flammability parameters of contemporary and historic oak under two imposed heat fluxes; (ii) to identify microstructural features that can plausibly affect local heating and ignition; (iii) to determine whether SEM–EDS reveals chemical heterogeneity in degraded and charred zones; and (iv) to formulate a conservation-relevant interpretation that distinguishes ignition sensitivity from total fire contribution without overstating causality beyond the experimental evidence.

## 2. Materials and Methods

### 2.1. Materials and Sample Origin

The contemporary material was oak wood (QN; *Quercus robur* L.) without visible macroscopic defects (Table 1). The historic material was oak wood obtained from farm no. 5 in Swołowo, Poland, from a half-timbered residential building dated to 1869. The material was acquired during revitalisation works, which enabled destructive testing without removing sound heritage material solely for research purposes [1]. Historic specimens were separated according to surface condition into non-degraded historic oak (QHND) and degraded historic oak (QHD). The degraded group contained deep cracks, losses, rough damaged surfaces and local disturbances of fibre arrangement.

### 2.2. Fire Testing in a Mass Loss Calorimeter

Specimens were prepared as mini-panels of 38 × 100 × 100 mm ± 1 mm, with the last dimension oriented along the grain. Six contemporary and twelve historic specimens were included in the fire-test design. After drying, weighing and measuring, the samples were tested in a mass loss calorimeter according to ISO 13927 and the procedure described by Clausen et al. [15,16]. The specimens were exposed to heat fluxes of 35 and 50 kW/m^2^ at a distance of 25 mm from the specimen surface; the exposed area was 88.4 cm^2^. The apparatus was calibrated with a methane burner before testing and after each change in heat-flux intensity [17].

The measured or calculated parameters were mass loss (ML), mass loss rate (MLR), heat release rate (HRR), total heat release (THR), effective heat of combustion (EHOC), time to ignition (TTI) and time to flame extinction (TTF). In the present manuscript, the MLC results were interpreted together with SEM and SEM–EDS observations because surface morphology and chemical heterogeneity can strongly influence the early stages of ignition.

**Figure 1:**
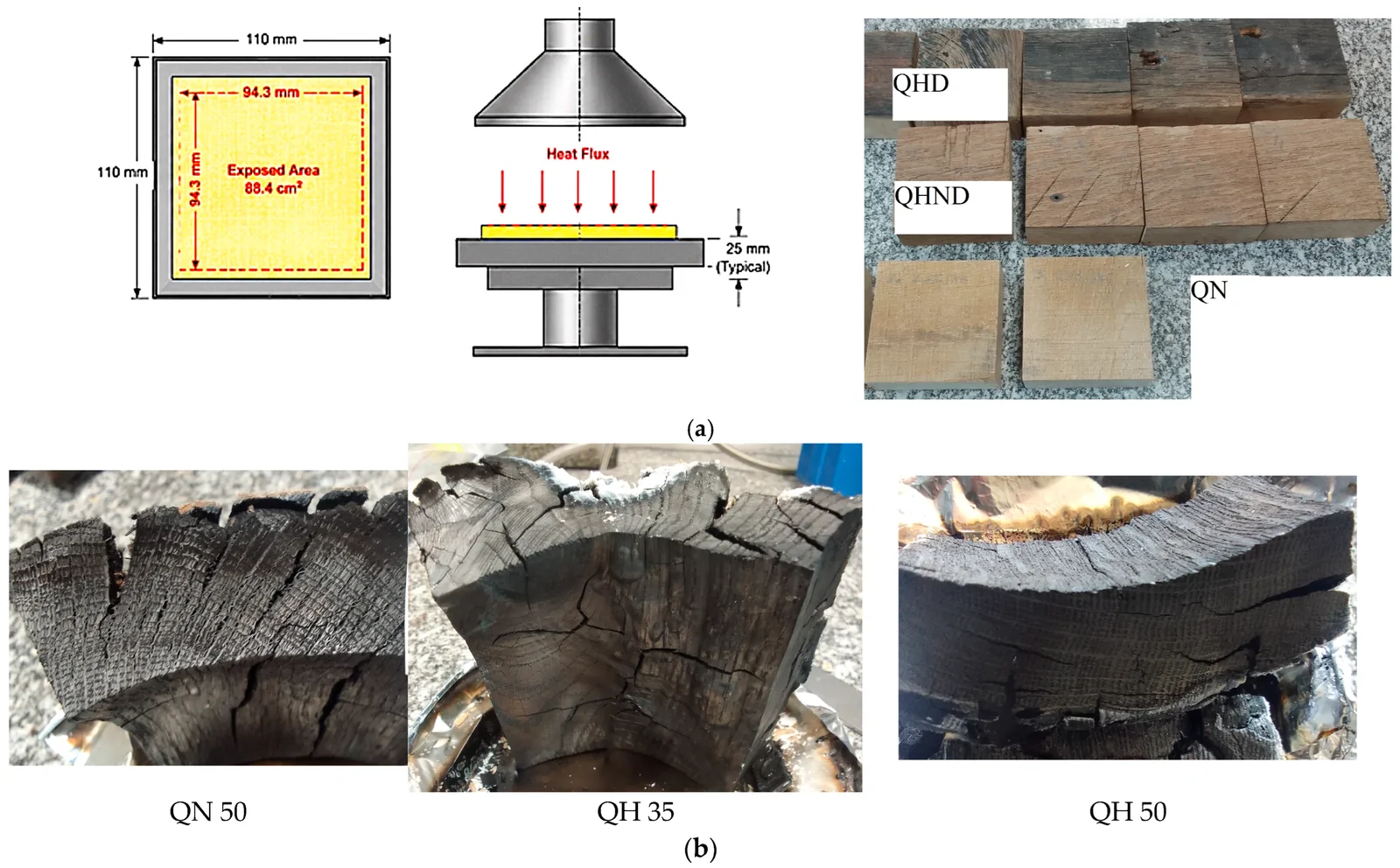
Flowchart of the testing process [15]. Sample specimens for testing (**a**) and after the test (**b**).

Only samples of historic wood with non-degraded surfaces were used for the tests at a heat flux of 35 kW/m^2^, due to the availability of the test material. Consequently, we decided to test degraded historic wood at a heat flux of 50 kW/m^2^, under more severe conditions (Figure 1).

### 2.3. SEM Microstructural Observations

Representative fragments of contemporary, aged non-charred, degraded aged and charred historic oak were examined by SEM. Specimens did not exceed approximately 10 mm in diameter and 3 mm in thickness. Each fragment was fixed to a specimen stub with an adhesive, and conductive tape was applied between the specimen edge and the stub to improve charge dissipation. The transverse sections analyzed in this study were not conductively coated. The coating status of the archived longitudinal and charred fragments could not be confirmed from the surviving preparation record and was therefore not used in the interpretation of elemental concentrations.

Transverse fields were used to assess vessel geometry and cell-wall continuity, whereas longitudinal fields were used to evaluate fibre continuity, fibrillation, fragmentation and thermally altered surface relief. The images represent local surface or near-surface fields and are not intended to characterize the entire specimen volume. Observations were qualitative and comparative rather than stereological. The assessed descriptors included anatomical readability, vessel and cell-wall continuity, surface relief, fibrillation, microcracking, delamination, apparent open porosity and particulate deposits. Anatomical terminology followed established IAWA descriptors [18].

SEM imaging was performed in the laboratory of the Faculty of Civil Engineering and Architecture, Lublin University of Technology, using a JEOL JSM-6300 scanning electron microscope (JEOL Ltd., Tokyo, Japan). The metadata of the representative micrographs document high-vacuum operation at 10 kV. Working distances were 9.3 mm at 100×, 9.0 mm at 200× and 9.9 mm at 500×; the corresponding horizontal field widths were 2.98 mm, 1.49 mm and 597 µm. Interpretation was based on the scale bars and actual fields of view rather than on the nominal maximum magnification of the microscope.

### 2.4. SEM–EDS Elemental Mapping

Energy-dispersive X-ray microanalysis was performed using a LINK–ISIS electron microprobe/EDS system (JEOL Ltd., Tokyo, Japan) coupled to the JEOL JSM-6300 microscope in the laboratory of the Faculty of Civil Engineering and Architecture, Lublin University of Technology. SEM–EDS analyses were conducted at 20 kV with a beam current of approximately 10^−9^ A and an acquisition time of 15 min per micro-area. The electron beam was directed at a selected point or rastered over a selected surface field to obtain localized spectra and elemental distribution maps. The nominal spatial resolution of the elemental analysis was approximately 1 µm, although the effective X-ray interaction volume depends on matrix density, composition, topography and accelerating voltage.

SEM–EDS was used to compare the local elemental signature of preserved, degraded and charred zones. C and O were considered the principal elements of the lignocellulosic matrix, whereas Na, Mg, Al, Si, P, S, Cl, K, Ca, Ti and Fe were evaluated as indicators of local inorganic heterogeneity. “Elemental enrichment” denotes a stronger or more extensive local signal relative to preserved fields within this dataset and does not imply enrichment throughout the complete timber member.

Elemental maps were interpreted jointly with the corresponding SEM field. Co-localization was accepted only when coherent elemental distributions corresponded to a visible particle, deposit, crack margin, wall fragment or altered zone and extended beyond isolated pixels. Co-occurrence was not treated as compound identification. Accordingly, Na–Cl, Ca–S, Si–Al and Fe–O associations were considered compatible with chloride-related residues, calcium- and sulphur-bearing deposits, aluminosilicate particles and oxidized Fe-rich material, respectively, but confirmation would require ion chromatography, FTIR, Raman spectroscopy or XRD.

The reported EDS concentrations are field-specific and semi-quantitative. Surface relief, pore geometry, interaction volume, detector geometry, counting statistics, peak overlap and matrix corrections can affect apparent intensities, particularly on rough and porous charred surfaces. The absence of coating on transverse sections excludes carbon or gold coating as a direct source of signals in those fields. Nevertheless, C, O and low-level N values were not interpreted as bulk composition or direct measures of oxidation and carbonization. Archived EDS working distance, detector take-off geometry, dead time, map pixel dimensions and matrix-correction settings were not retained in the available documentation; the results are therefore used comparatively rather than as fully traceable bulk quantitative analyses.

### 2.5. Data Interpretation, Reproducibility and Reviewer-Safe Assumptions

The MLC data are reported as mean values with standard deviations, but the experimental design was not treated as a full inferential statistical model because of the limited number of historic specimens, the heterogeneity of the source material and the condition-based grouping of surfaces. Consequently, the interpretation focuses on robust directional differences, mechanistic consistency and agreement between independent observations rather than on overextended statistical claims. This approach is appropriate for heritage materials, where destructive sampling is intrinsically constrained and the variability of historic fabric is part of the research object (Table 2).

SEM observations were interpreted as qualitative microstructural evidence. Terms such as “higher roughness”, “increased fibrillation” and “secondary porosity” describe comparative visual trends visible in the supplied fields of view; they should not be read as calibrated roughness, porosity or crack-density measurements. SEM–EDS values are reported as field-specific semi-quantitative elemental results and should not be extrapolated to the whole timber member without additional sampling. Elemental detection was intentionally separated from phase identification because SEM–EDS cannot unambiguously identify salts, oxides or mineral compounds.

## 3. Results

### 3.1. Flammability Parameters Obtained in MLC Tests

The average MLC results are summarized in Table 3. The HRR curves followed the expected course for wood: after ignition, the heat release rate increased, then decreased as a char layer developed, and in some cases increased again when the char layer degraded or cracked. This behaviour is consistent with the temporary protective role of char as a thermal barrier.

**Figure 2:**
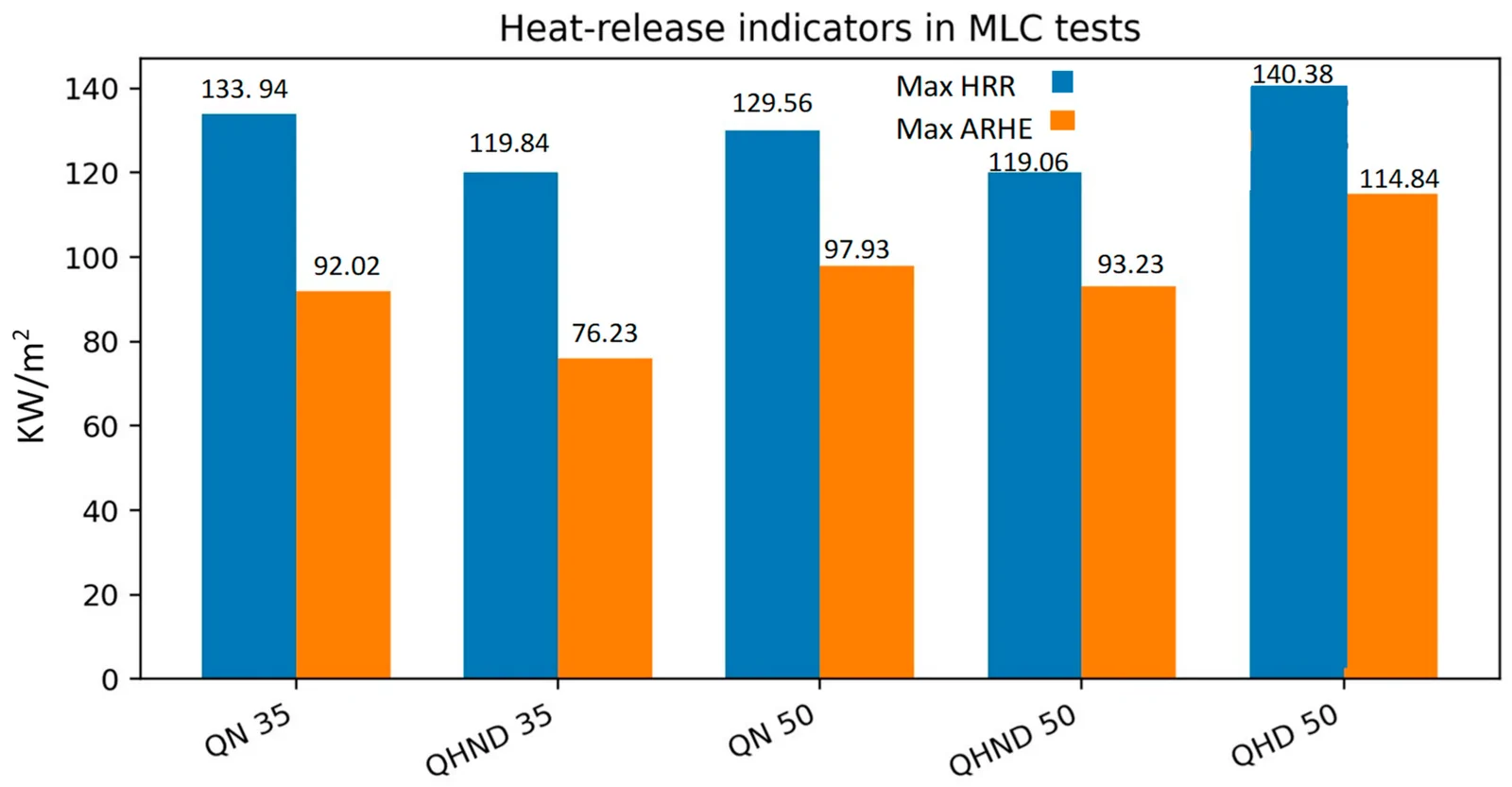
Comparison of maximum HRR and maximum ARHE in the MLC tests.

Non-degraded historic oak showed lower maximum HRR than contemporary oak at both heat fluxes. At 35 kW/m^2^, the maximum HRR decreased from 134 kW/m^2^ for QN to 120 kW/m^2^ for QHND. At 50 kW/m^2^, it decreased from 130 kW/m^2^ for QN to 120 kW/m^2^ for QHND. The degraded historic variant at 50 kW/m^2^ reached 140 kW/m^2^, indicating that strongly damaged surfaces can locally increase early heat-release indicators under stronger heat flux (Figure 2).

**Figure 3:**
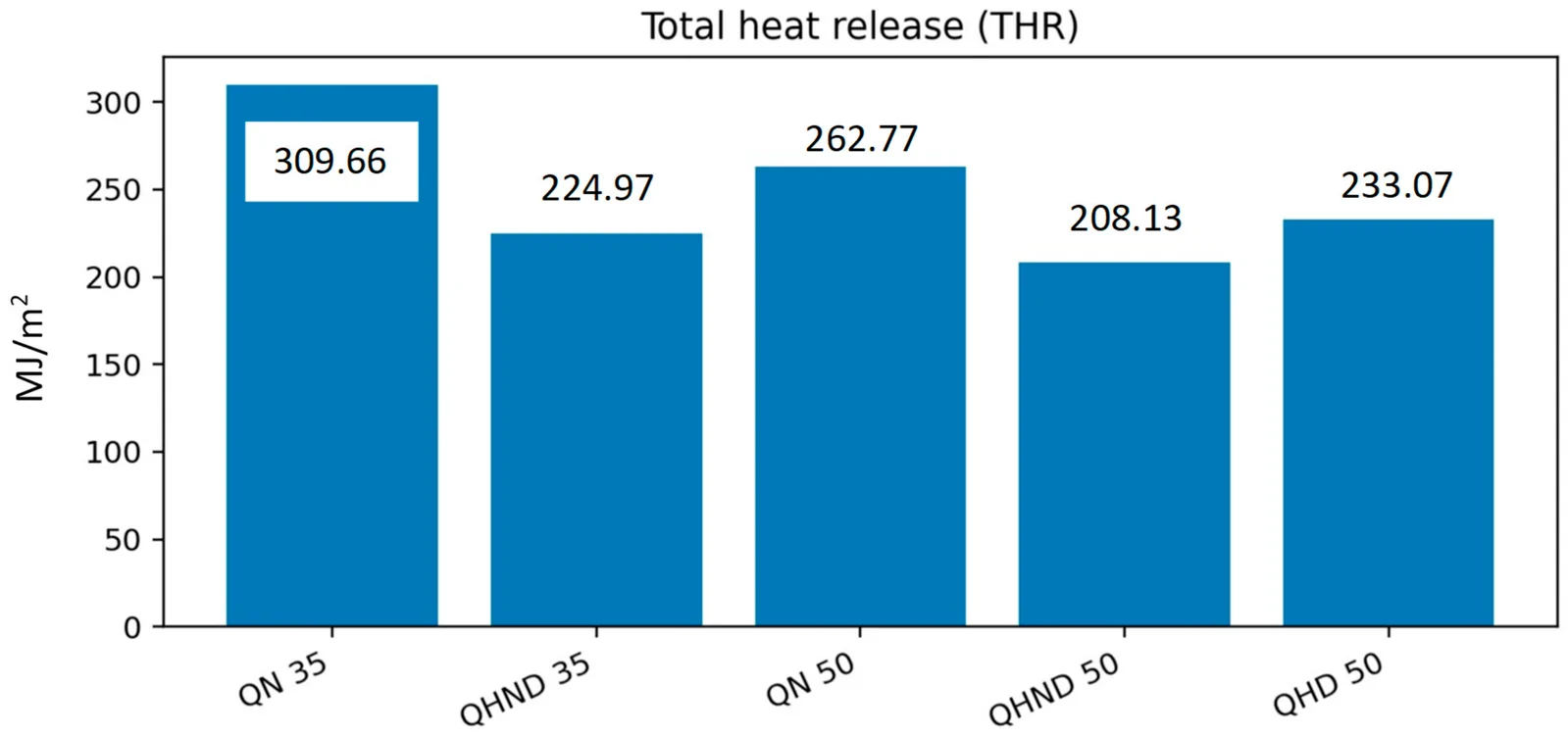
Total heat release (THR) in contemporary and historic oak variants.

**Figure 4:**
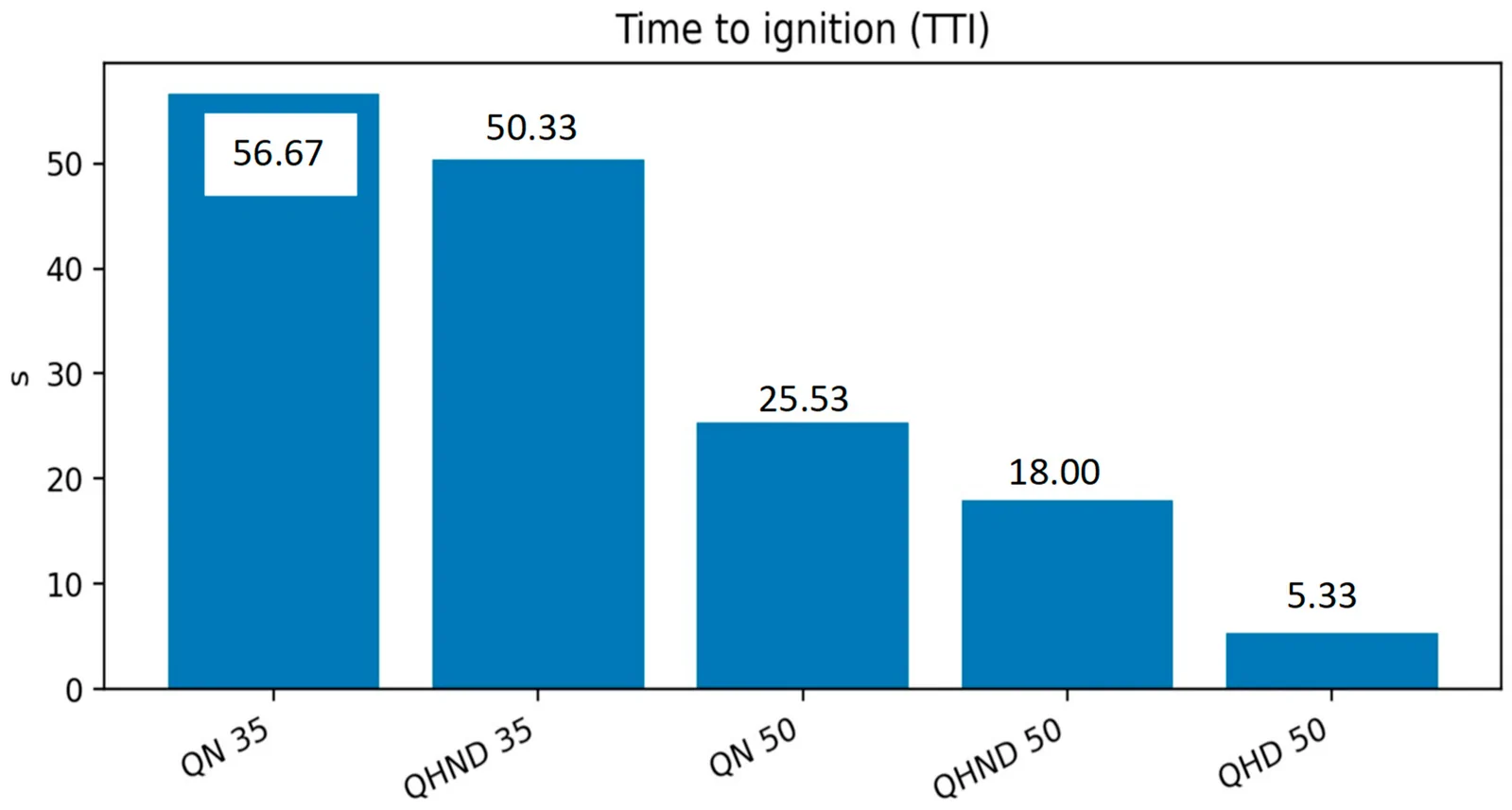
Time to ignition (TTI) in contemporary and historic oak variants.

Total heat release was lower for historic variants than for contemporary oak in the directly comparable cases. At 35 kW/m^2^, THR decreased from 310 MJ/m^2^ for QN to 225 MJ/m^2^ for QHND. At 50 kW/m^2^, THR decreased from 263 MJ/m^2^ for QN to 208 MJ/m^2^ for QHND and 233 MJ/m^2^ for QHD (Figure 3). Final mass loss remained similar, approximately 73–74%, in all variants. Therefore, the main differences concerned the dynamics of burning rather than the final degree of material consumption. Time to ignition (TTI) varied considerably among the analyzed samples (Figure 4). The highest ignition resistance was observed for the QN 35 specimens, which exhibited the longest TTI of 56.67 ± 8.3 s, followed by QHND 35 with 50.33 ± 5.3 s. Increasing the heat flux from 35 to 50 kW/m^2^ resulted in a pronounced reduction in ignition time. For the untreated material (QN 50), TTI decreased to 25.33 ± 2.4 s, whereas QHND 50 ignited after 18.00 ± 3.6 s. The shortest ignition time was recorded for QHD 50, reaching only 5.33 ± 0.9 s, indicating a very rapid ignition under the applied test conditions. Overall, the results demonstrate that increasing the external heat flux substantially accelerates ignition, while differences between the tested wood variants become more pronounced at higher thermal exposure. The approximately tenfold difference between QN 35 and QHD 50 highlights the strong influence of test conditions and material characteristics on ignition behaviour.

### 3.2. SEM-Based Microstructural Characterization

**Figure 5:**
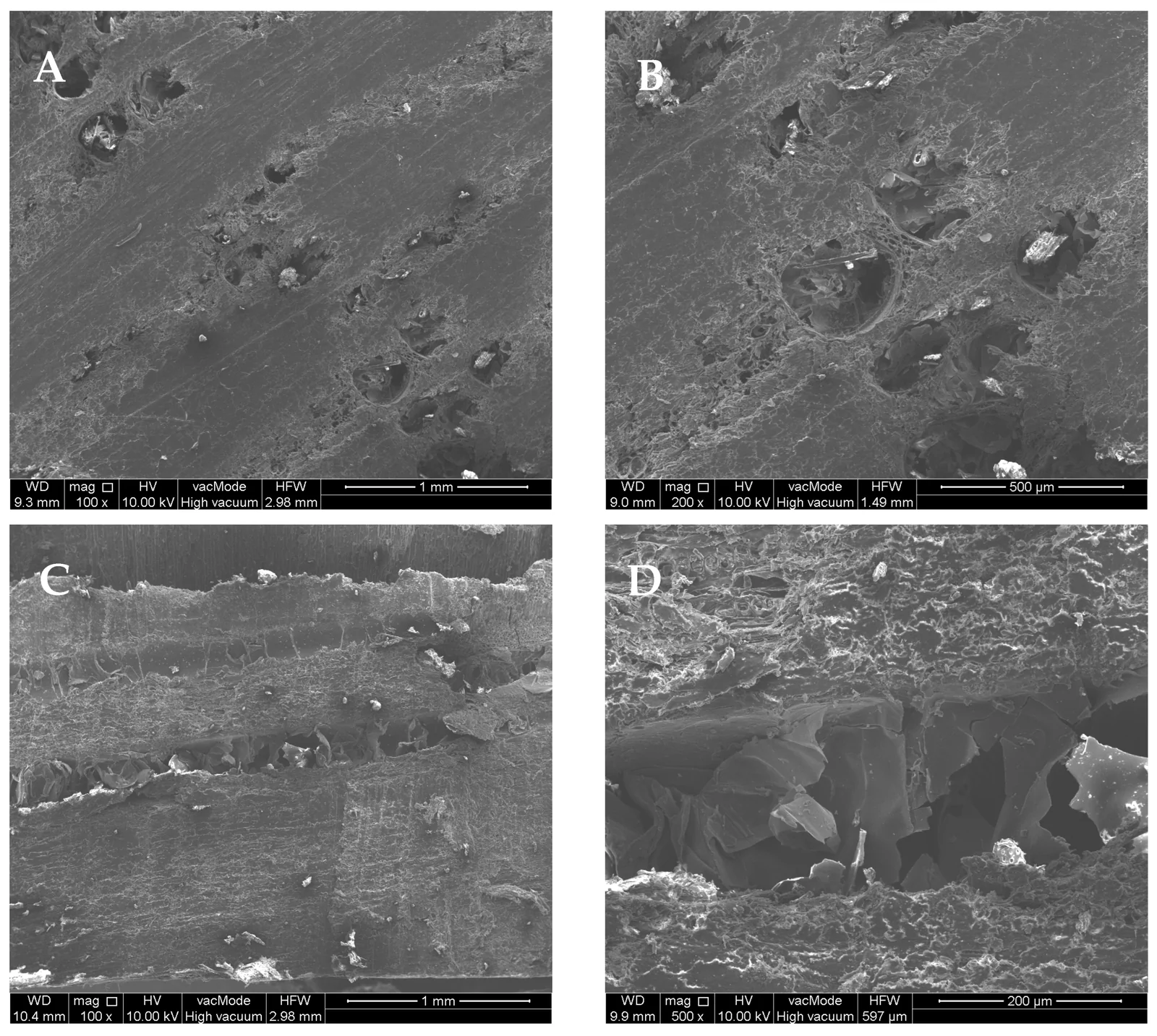
SEM micrographs of contemporary oak wood. Panels (**A**,**B**): transverse section; panels (**C**,**D**): longitudinal section. Scale bars are embedded in the original SEM images.

**Figure 6:**
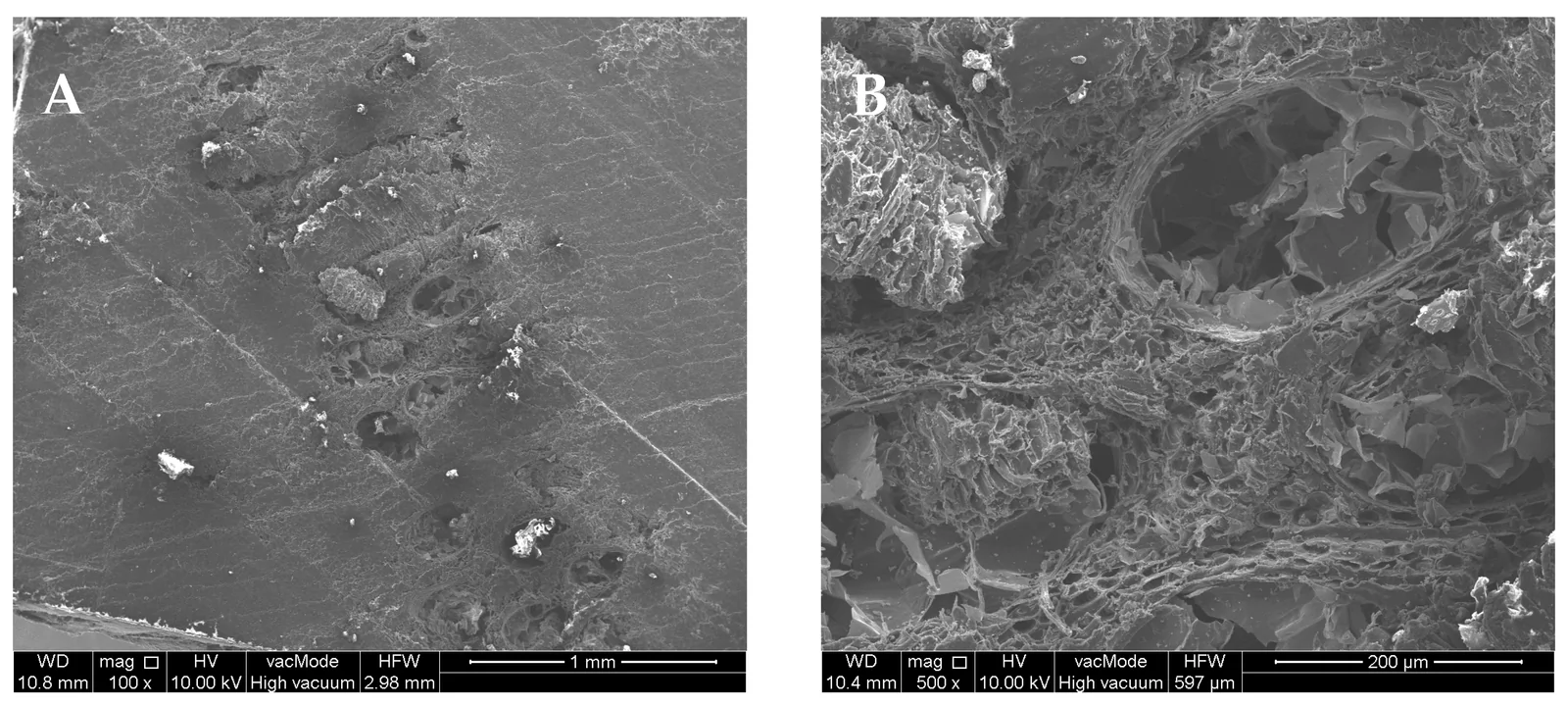

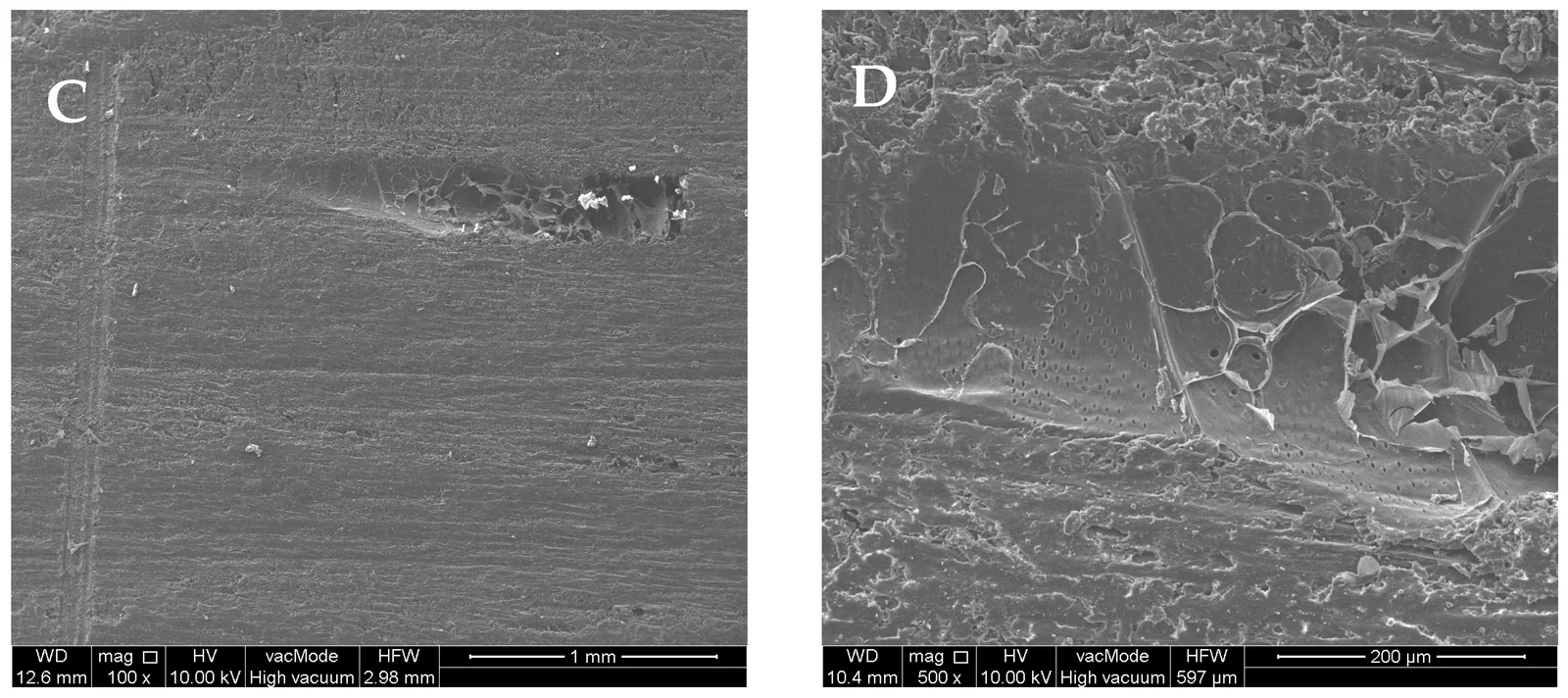
SEM micrographs of aged oak wood. Panels (**A**,**B**): transverse section; panels (**C**,**D**): longitudinal section. Scale bars are embedded in the original SEM images.

**Figure 7:**
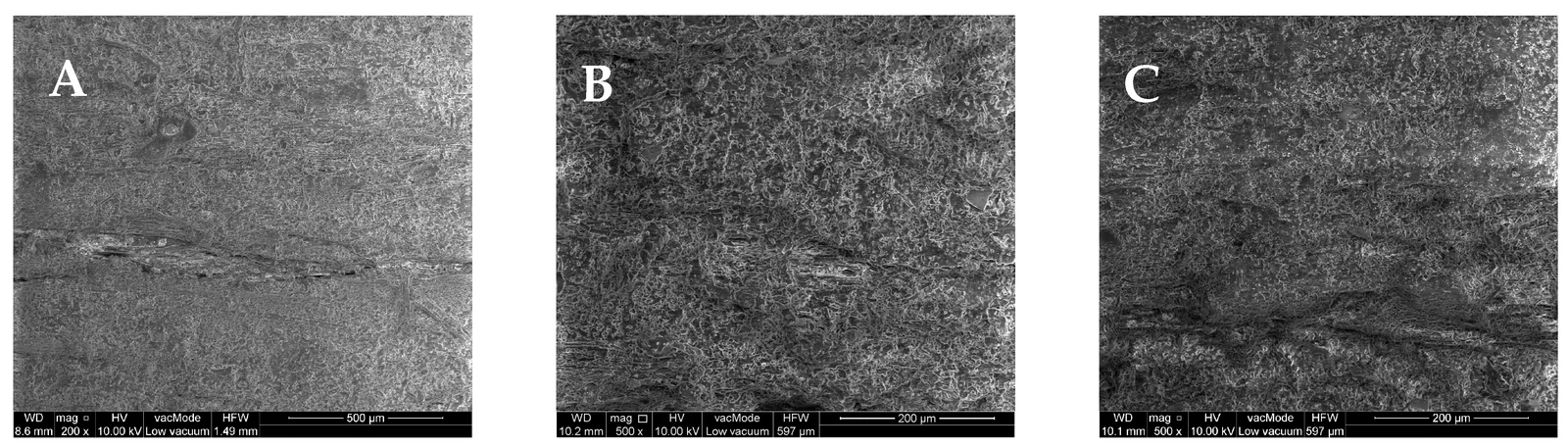

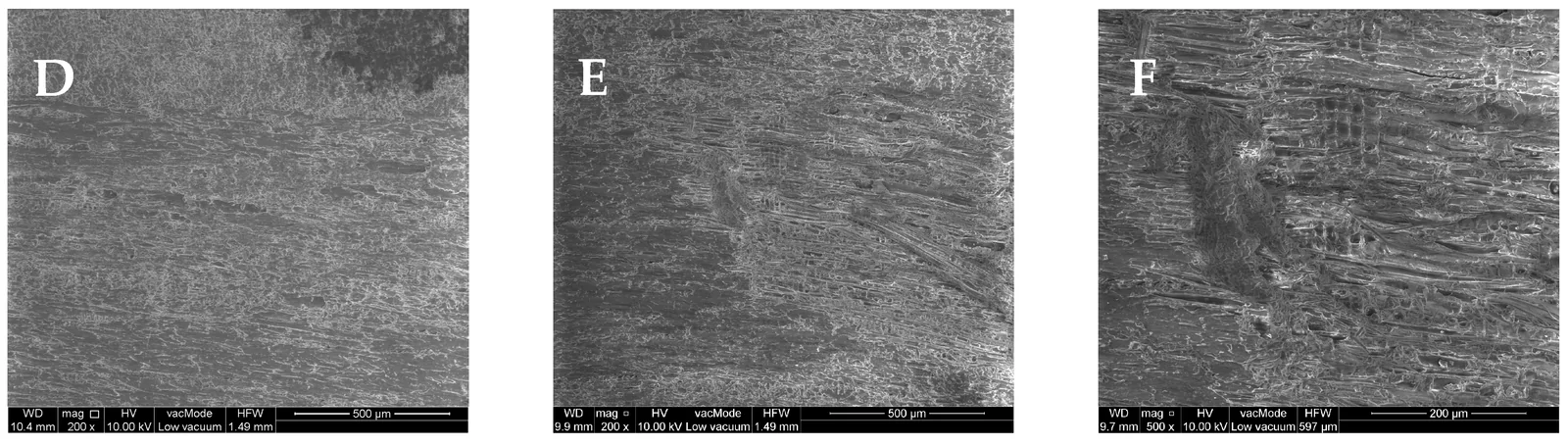
SEM micrographs of thermally affected and damaged aged oak. Panels (**A**–**C**): charred wood in longitudinal section; panels (**D**–**F**): damaged aged wood in longitudinal section. Scale bars are embedded in the original SEM images.

SEM observations showed clear differences in surface and near-surface preservation between contemporary, aged and charred oak. Contemporary oak retained comparatively regular and readable anatomy, with visible vessel lumina, continuous cell-wall boundaries and a compact matrix surrounding anatomical openings, consistent with the hierarchical organization of wood tissues and cell walls [19,20]. Aged oak showed more heterogeneous relief, locally interrupted wall continuity, fractured edges, fibrillation and attached particles or deposits. These features were interpreted as spatially heterogeneous local alteration rather than uniform degradation of the complete tissue volume (Figure 5, Figure 6 and Figure 7).

The contemporary reference material showed the highest anatomical readability. Vessel lumina and cell-wall boundaries were clearly identifiable, and preparation-related tearing did not dominate the fields. Aged oak retained the general oak anatomical pattern but exhibited uneven relief, local delamination, fractured wall edges and fibrillated fragments. These observations are compatible with local weakening of the near-surface cellular network and agree with microscopy-based descriptions of degraded archaeological and historic wood [21,22,23]. The terms “roughness” and “porosity” are used qualitatively; no profilometric roughness or stereological pore-volume measurement was performed (Table 4).

Charred historic oak displayed the most advanced surface alteration. Fibre orientation remained partly recognizable, but the near-surface zone contained jagged cavity margins, microvoids, discontinuous wall remnants and plate-like or lamellar fragments separated by open discontinuities. The morphology is compatible with local delamination, brittle fragmentation and secondary material loss [20,24]. Some observed voids may include anatomical openings or preparation-related losses; therefore, they were not treated as a quantitative measure of porosity.

### 3.3. SEM–EDS Elemental Mapping

In the relatively preserved historic fields, SEM–EDS spectra were dominated by C and O, consistent with an organic lignocellulosic matrix [20]. Two preserved longitudinal fields contained 53.79 and 58.55 wt.% C and 45.04 and 40.64 wt.% O, respectively. In the analyzed transverse field, C was 66.88 wt.% and O 32.16 wt.%, with minor K and trace Na. These are normalized local results, not bulk wood chemistry. Differences in C/O may partly reflect the local proportion of wall material, lumen edges and deposits within the X-ray interaction volume; the values were therefore not used to calculate oxidation or degradation rates.

**Figure 8:**
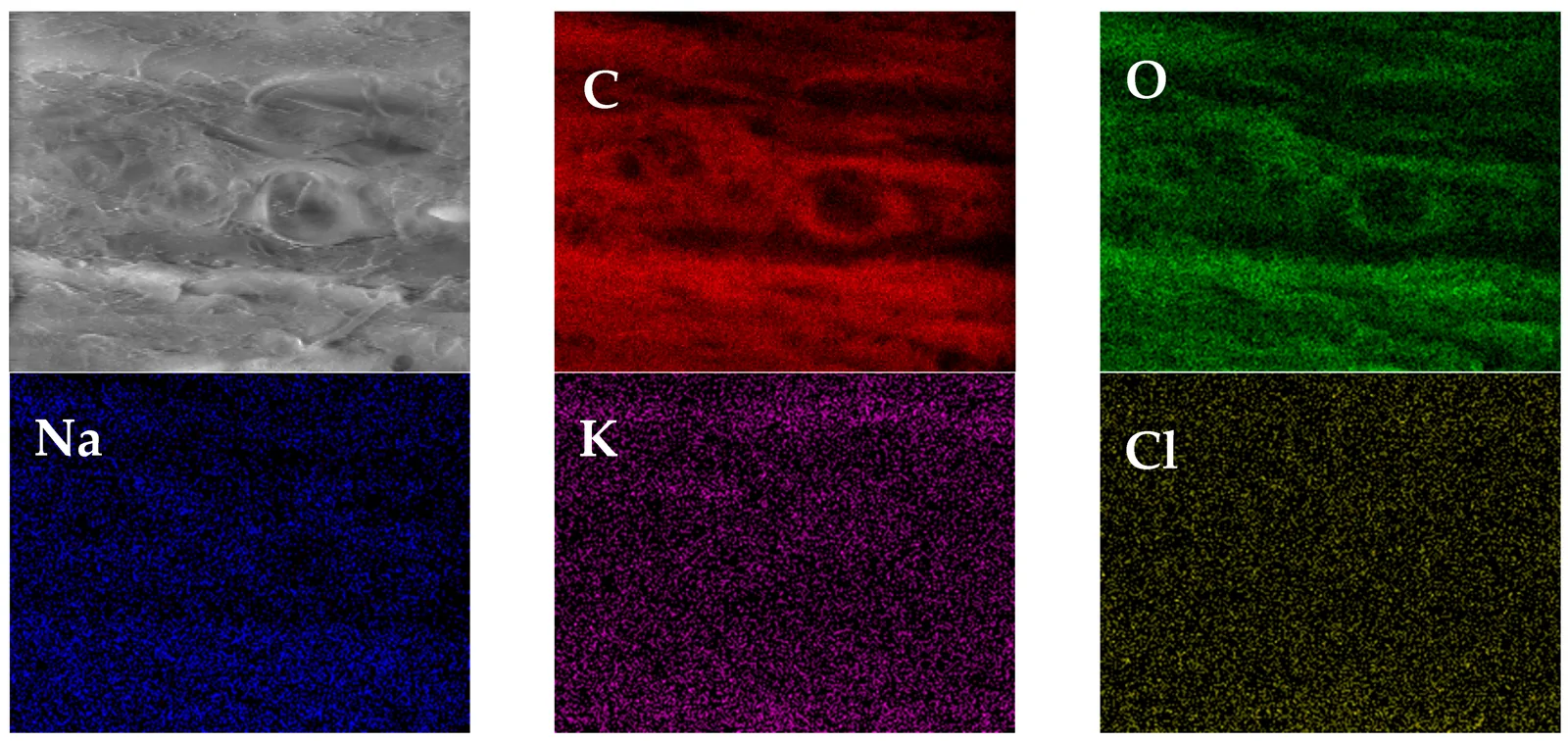
SEM–EDS maps of aged oak wood in longitudinal section. The panels show the SEM field and elemental distributions of C, O, Na, K and Cl.

The preserved fields contained only spatially limited low-intensity signals of Na, K and Cl relative to C and O (Figure 8). The distributions do not demonstrate a continuous salt phase and are interpreted only as discrete or weak inorganic residues in an otherwise organic-matrix-dominated local field.

**Figure 9:**
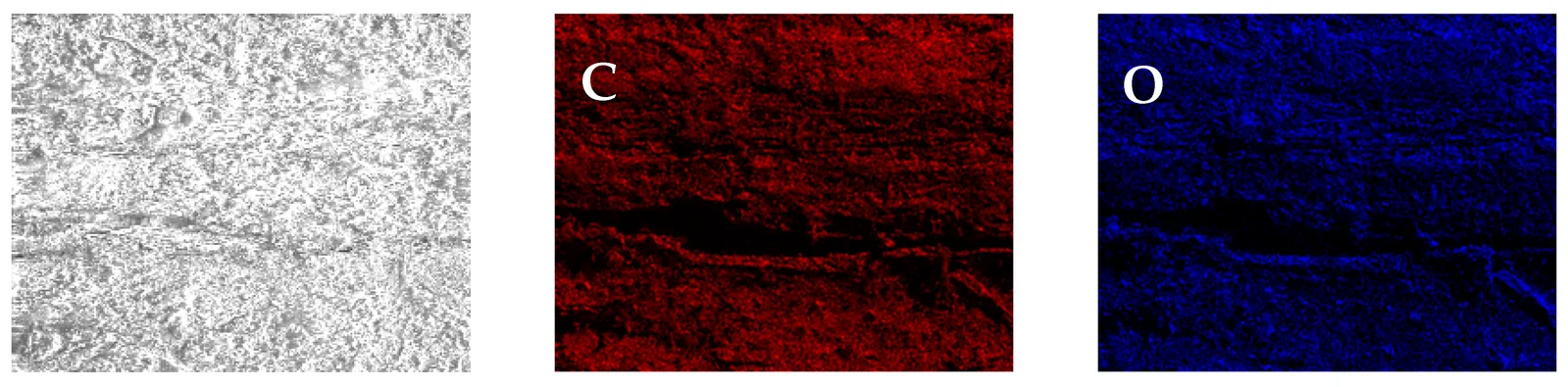

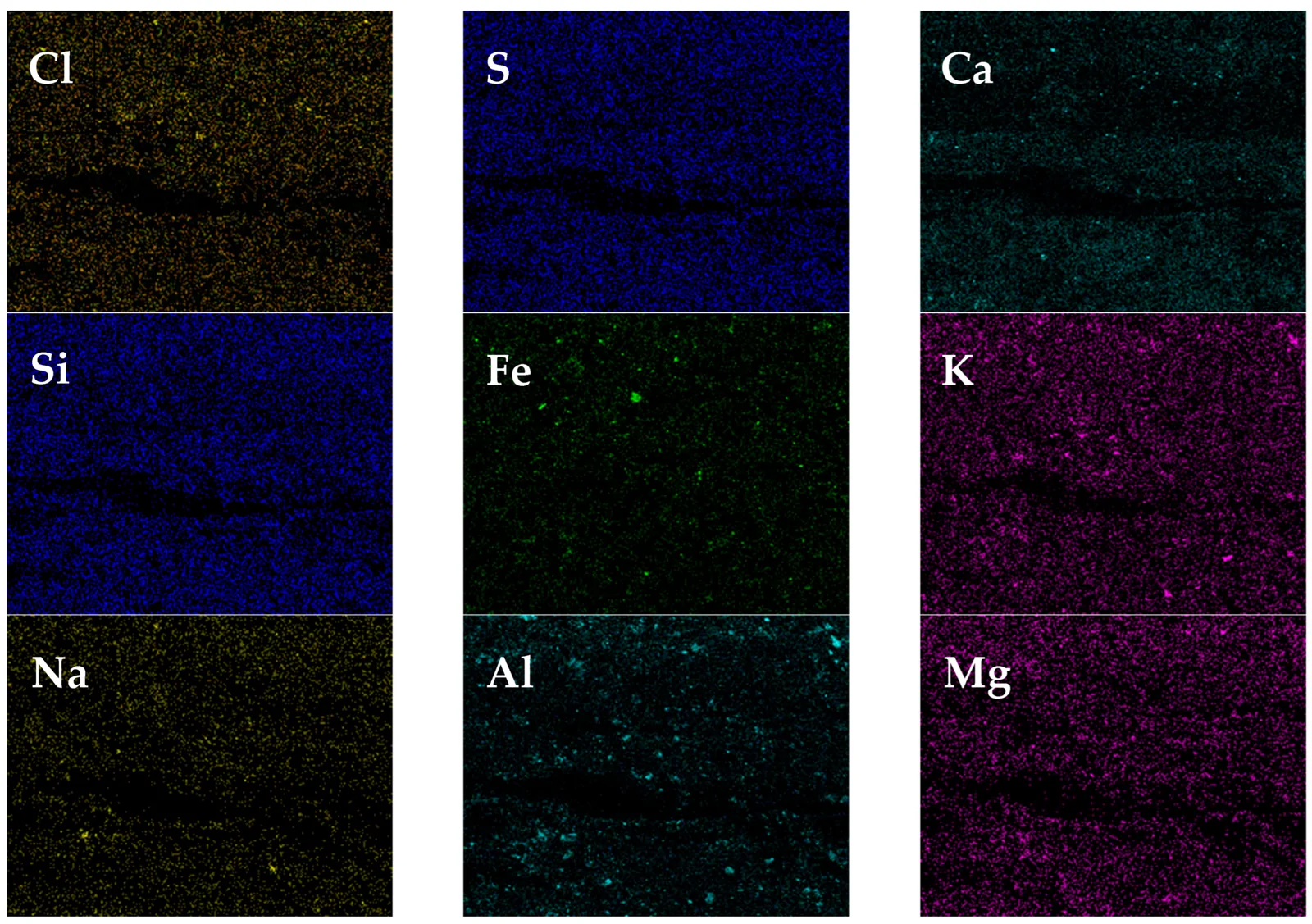
SEM–EDS maps of charred historic oak wood in longitudinal section. The panels show the SEM field and selected elements detected in the degraded or charred surface region.

Degraded and charred fields showed a broader local elemental spectrum comprising C, O, N, Na, Mg, Al, Si, P, S, Cl, K, Ca, Ti and Fe (Figure 9). Inorganic signals were spatially heterogeneous and tended to occur in discrete deposits, cavities, crack margins and fragmented surface zones rather than uniformly throughout the wood matrix. This pattern is consistent with the SEM morphology, because surface opening and secondary porosity can provide retention sites for mineral particles and mobile ions.

The element groups were interpreted conservatively (Table 5). Spatial association of Si and Al is compatible with aluminosilicate or mineral-dust particles but does not distinguish soil-derived particles from construction dust. Ca may reflect contact with lime-bearing mortar or plaster, while local Ca–S co-occurrence is compatible with a calcium- and sulphur-bearing deposit. Fe-rich particles may be associated with contact with metal or corrosion-derived material, but Fe alone does not identify a specific oxide or hydroxide. Na, K, Mg, Cl and S may indicate mobile ionic or salt-related residues; their mineral form, hydration state and origin remain unresolved. Sparse Ti, P or N signals were not assigned to compounds without defensible peak and map evidence.

In charred zones, the broader elemental spectrum may reflect concentration of non-volatile ash-forming constituents during thermal conversion, external contamination retained by porous char, or both. The present data cannot separate these mechanisms quantitatively. The defensible conclusion is that degraded and charred fields were more chemically heterogeneous at the analyzed microscale than the preserved fields. No causal effect of an individual element on MLC parameters is claimed because compound identity, concentration and distribution were not established.

## 4. Discussion

### 4.1. Relationship Between Surface Degradation and Ignition

The clearest difference between contemporary and historic oak was the time to ignition. At 35 kW/m^2^, QHND ignited after 50.33 s compared with 56.67 s for QN. At 50 kW/m^2^, QHND ignited after 18 s compared with 25.33 s for QN. The degraded historic variant ignited after only 5.33 s at 50 kW/m^2^. This trend is consistent with SEM observations: rough surfaces, fibrillated wall fragments, cracked cell-wall edges and loose particles create thin, exposed and thermally sensitive points that can reach pyrolysis and ignition conditions faster than compact tissue.

However, faster ignition should not be equated with poorer global fire performance. Ignition describes the start of flaming under a defined external heat flux, whereas HRR, THR and final mass loss describe subsequent heat-release and degradation processes. In the available data, historic wood ignited faster but generally did not produce higher THR or final mass loss. This distinction is essential in conservation decisions because a material can be ignition-sensitive at the surface while still showing comparable or lower total heat contribution.

### 4.2. Heat Release, Char Formation and Total Fire Contribution

The non-degraded historic variants showed lower maximum HRR and lower THR than contemporary oak. The degraded historic variant at 50 kW/m^2^ was an exception for maximum HRR and ARHE, indicating that severe surface degradation can intensify the early heat-release response. Nevertheless, THR remained lower for QHD 50 than for contemporary oak at the same heat flux. Similar final mass loss values of approximately 73–74% suggest that the final degree of material consumption was comparable under the selected test conditions.

These results support a two-stage interpretation of historic oak behaviour under external heat flux. In the first stage, roughness, cracks, thin fibrils and detached particles reduce local thermal inertia and accelerate ignition. In the second stage, heat release is controlled by density, available combustible mass, moisture content, heat transfer into the specimen and char-layer formation. A charred layer may act as a temporary thermal barrier, but its function depends on continuity, cohesion and resistance to detachment. SEM images of charred wood show that this layer can be porous, fragmented and mechanically vulnerable.

### 4.3. Microstructural Opening and Mineral Enrichment

The combined SEM and SEM–EDS observations indicate an association between microstructural opening and local elemental heterogeneity [20,21,22,23]. Cracks, delaminated fragments, secondary cavities and porous char provide plausible pathways or retention sites for particles and ions. However, the direction of causality cannot be determined from these observations: deposits may accumulate after surface disruption, while hygroscopic or crystallizing residues may also contribute to subsequent damage during environmental cycling.

From a conservation perspective, the analytical sequence should proceed from spatial screening to phase-specific identification. SEM–EDS can locate deposits and define relevant element groups; ion chromatography can quantify soluble ions; FTIR or Raman spectroscopy can identify molecular groups; and XRD can identify crystalline phases. Bulk ash or ICP-based analysis would be needed to distinguish the overall inorganic inventory from isolated surface particles. The present study does not equate elemental detection with salt or mineral identification.

The possible relationship between inorganic residues and fire behaviour remains a testable hypothesis. Alkali and alkaline-earth compounds may affect dehydration and char-forming reactions, while mineral particles may modify local heat transfer. Conversely, some residues may be inert under ignition conditions. Because compound identity and concentration were not established, no catalytic, fire-promoting or fire-retarding role is assigned to individual elements. The observed reduction in TTI is linked primarily to documented surface morphology, with elemental heterogeneity treated as a potential modifying factor requiring controlled experiments.

Accordingly, the detected Si, Al, Ca, Fe, Na, K, Mg, Cl and S signals are discussed as provenance and conservation indicators rather than as identified compounds. Representative deposits should be verified by complementary phase-specific methods before cleaning, consolidation or fire-retardant treatment is selected for chemically heterogeneous historic surfaces.

### 4.4. Conservation and Fire-Safety Implications

The present results have direct implications for the conservation and fire-safety assessment of historic timber because they demonstrate that ignition sensitivity and cumulative fire contribution are not interchangeable descriptors. Relative to contemporary oak, the non-degraded historic oak ignited 11.2% earlier at 35 kW/m^2^ and 28.9% earlier at 50 kW/m^2^, while the degraded historic surface ignited 79.0% earlier than contemporary oak at 50 kW/m^2^. At the same time, the THR of QHND was 27.4% lower at 35 kW/m^2^ and 20.9% lower at 50 kW/m^2^, and QHD still showed an 11.4% lower THR than QN at 50 kW/m^2^. Final mass loss remained within the narrow range of 73–74%. Thus, the tested historic oak was more susceptible to rapid surface ignition, particularly when visibly degraded, but did not release more cumulative heat or undergo a greater final degree of consumption under the applied MLC conditions [25,26].

This separation between ignition and post-ignition heat release is consistent with several studies on naturally aged or historic wood. Chorlton and Gales reported shorter ignition times and charring rates up to approximately 20% higher for nineteenth-century softwood timbers than for contemporary glulam, although they also emphasized differences in density, growth-ring structure and material origin [9]. Wang et al. observed that ancient timbers from traditional Chinese buildings tended to ignite earlier and, for some species and surface conditions, exhibited higher peak heat release and less regular HRR curves than modern reference wood [27]. Liu et al. likewise demonstrated the strong dependence of ignition time on external heat flux for aged cypress and fir tested at 25, 35 and 50 kW/m^2^ [28]. These studies support the present conclusion that historic timber can be especially vulnerable during the initiation stage of fire, but they also show that the magnitude and direction of the response depend on species, density, surface morphology and test configuration.

The lower HRR and THR measured for the historic oak variants agree particularly well with results reported for naturally aged pine. Deng et al. found that approximately 100-year-old pine had a lower ignition temperature, enlarged pores and damaged cell structure, but also formed a relatively dense carbonized layer and produced lower HRR and THR than fresh pine [8]. Long et al., comparing Masson pine with service times of approximately 5, 170 and 400 years, reported that THR decreased by 16.0% and 34.7% in the two older groups relative to the youngest material, despite progressive microstructural disruption and reduced thermal stability [29,30]. This research provides an important analogue for the present oak results: long-term ageing or degradation may facilitate the onset of pyrolysis and ignition while simultaneously reducing the amount or rate of combustible volatile release during the later stages of burning. Nevertheless, species-specific chemistry and anatomical differences preclude direct numerical transfer from pine to oak.

Not all forms of ageing or surface change produce the same fire response. Pánek et al. observed pronounced colour change, surface roughening and lignin depletion in four naturally weathered softwoods, yet found no significant change in ignition or relative burning rate after up to 24 months of outdoor exposure [29]. This apparent contrast with the present results is scientifically relevant rather than contradictory. Short-term weathering of otherwise intact specimens is not equivalent to more than 150 years of service accompanied by cracking, material loss, fibrillation, contamination and local thermal damage. The comparison indicates that chronological exposure alone is an insufficient predictor; the intensity, duration and type of deterioration, together with the initial wood quality and subsequent service history, determine whether surface changes become fire-relevant.

For conservation practice, the most defensible approach is therefore condition-based and spatially resolved. Visual inspection should identify cracks, checks, losses, friable fibres, previous charring, deposits and interfaces with mortar, plaster or metal. These observations should be combined with moisture content, density, species identification and, where feasible, targeted microscopic or small-scale fire testing. The relevance of cracks is supported by Harun et al., who showed that pre-existing radial cracks in heritage pine expanded during fire exposure and produced markedly greater local char depths at crack locations, even though their effect on the global load-bearing capacity was more limited [31]. In the present material, degraded surface morphology similarly appears to define local ignition-sensitive zones rather than a uniform deterioration of the whole cross-section.

The practical consequence is not that exposed historic oak should automatically be removed, encapsulated or replaced. Such interventions may unnecessarily reduce authenticity and conceal diagnostically valuable surfaces. Instead, strongly degraded zones should be prioritized for risk reduction through removal of loose combustible debris where conservation ethics permit, compatible consolidation, control of ignition sources, improved detection, compartmentation and carefully selected passive or active fire-protection measures [2,25,26]. Well-preserved historic zones may remain exposed when their actual condition and the surrounding fire-safety strategy justify this decision. Because current non-destructive methods cannot yet predict the complete fire performance of reused or historic timber with adequate reliability, diagnostic evidence should inform but not replace engineering assessment [32].

### 4.5. Mechanistic Interpretation and Strength of Evidence

The combined MLC, SEM and SEM–EDS dataset supports a multi-stage mechanistic interpretation (Table 6). The first and strongest experimental driver was incident heat flux. Increasing the flux from 35 to 50 kW/m^2^ reduced TTI by 55.3% for contemporary oak and by 64.2% for non-degraded historic oak. This is consistent with established ignition models in which higher external radiant flux accelerates surface heating, drying and volatile generation and therefore shortens the time required to reach piloted ignition conditions [11,12,28]. The larger reduction observed for QHND suggests that the near-surface condition modifies the response to irradiance, but the present design does not permit a formal interaction effect to be isolated statistically.

Within a given heat-flux level, surface preservation state provides the most coherent explanation for the ignition sequence. SEM documented fibrillation, fractured wall edges, lamellar fragments, delamination and secondary voids in aged and damaged zones. These features can create thin elements with low local thermal inertia, increase the area directly exposed to radiant heating and oxygen, and provide pathways for hot gases and flames. The very short TTI of QHD at 50 kW/m^2^ (5.33 ± 0.9 s) is therefore physically consistent with its visibly disrupted surface. Independent studies provide convergent support: natural ageing has been associated with enlarged pores and cell-wall damage in pine [8,30], ancient wood with irregular surface morphology has shown earlier ignition [27], and radial cracks in heritage timber have altered local char-front development and intensified damage around the crack [31]. This convergence strengthens the mechanism, although it does not quantify the contribution of each individual morphological descriptor.

The HRR and THR results indicate that the mechanisms governing ignition did not control the entire burning history. For QHND, maximum HRR was 10.4% lower than QN at 35 kW/m^2^ and 7.7% lower at 50 kW/m^2^, while THR was lower by 27.4% and 20.9%, respectively. QHD behaved differently at 50 kW/m^2^: maximum HRR increased by 7.7% and maximum ARHE by 17.3% relative to QN, yet THR remained 11.4% lower. This combination is compatible with a short, intense early response from a highly disrupted surface followed by a lower cumulative energy release. Similar decoupling has been reported for naturally aged pine, where earlier thermal degradation coexisted with reduced HRR or THR because of changes in combustible content, heat transfer and char formation [8,30]. The present results therefore extend this concept to historic oak and, importantly, distinguish non-degraded from visibly degraded historic surfaces.

The nearly identical final mass loss of 73–74% provides additional evidence that the principal differences concerned the rate and temporal distribution of combustion rather than the final fraction of material consumed. Final mass loss alone would therefore fail to identify the very large difference in TTI between QN and QHD. Conversely, peak MLR should be interpreted cautiously: the QN 50 group showed a value of 1.19 ± 1.3 g/s, with variability exceeding the mean, whereas the remaining groups were clustered around 0.20–0.26 g/s. This dispersion weakens any mechanistic inference based on peak MLR and reinforces the use of the more stable combination of TTI, HRR, THR and final mass loss. More generally, previous work has shown that mass loss rate, charring rate and structural resistance are related but non-equivalent quantities [13,14,32].

Density, moisture content, species and chemical composition remain important competing explanations. Moisture was comparable across the present groups (7–8%), reducing but not eliminating its confounding effect. Density, however, ranged from approximately 580 to 720 kg/m^3^ in the historic specimens compared with approximately 737 kg/m^3^ for contemporary oak. Studies across wood species generally associate density with ignition, heat transfer and charring, but the relationships are not universally monotonic and can be modified by extractives, permeability and anatomical orientation [11,33,34]. Haurie et al. further showed that hardwood fire behaviour reflects an interaction between density, morphology, mineral content and extractives rather than a single dominant parameter [33]. Consequently, the lower THR of the historic groups cannot be attributed uniquely to ageing, surface damage or density. Density-matched specimens and multivariable statistical designs would be required to separate these effects.

The SEM–EDS results provide a lower level of mechanistic evidence than the MLC and SEM observations. The broader occurrence of Na, Mg, Al, Si, P, S, Cl, K, Ca, Ti and Fe in degraded or charred fields demonstrates local chemical heterogeneity, but not the identity, concentration or fire function of specific compounds. Minerals and extractives can influence pyrolysis and char yield [33], yet the direction of their effect depends on chemical form, loading and distribution. The present data therefore support only the hypothesis that inorganic residues may modify local heat transfer or decomposition. They do not support assigning a catalytic, fire-promoting or fire-retarding role to any detected element. Phase-specific analysis and controlled treatment experiments are necessary before such causal claims can be made.

The strength of evidence can consequently be ranked. The evidence is strong within this dataset for (i) the reduction in TTI with increasing heat flux; (ii) the earlier ignition of historic, especially degraded, surfaces; (iii) the lower THR of all tested historic variants relative to contemporary oak under comparable flux; and (iv) the similarity of final mass loss. Evidence is moderate and associative for the role of roughness, cracks, fibrillation and secondary porosity, because the SEM observations are local and qualitative. Evidence is weak and hypothesis-generating for the influence of individual inorganic constituents. Cross-study agreement with historic and naturally aged timber research [8,9,27,28,29,30,31,32] increases external plausibility, whereas differences in species, density, orientation, specimen geometry, ageing history and calorimetric protocol limit direct quantitative comparison.

The main scientific contribution of the study is therefore not a universal assertion that old wood is more or less combustible than new wood. Rather, it is the demonstration, within one hardwood species and a defined heritage source, that preservation state differentiates ignition behaviour and that early ignition can coexist with lower cumulative heat release. Integrating MLC parameters with SEM morphology and conservative SEM–EDS interpretation provides a condition-based framework that is more informative than chronological age alone. This framework is compatible with current reviews, which conclude that no single measurable property or non-destructive test can yet predict the fire performance of reused timber and that combined evidence from species, density, moisture, geometry and service-induced defects is required [32,35].

## 5. Study Limitations and Future Research

The SEM and SEM–EDS component has several limitations. The microscopic observations are local and qualitative and should not be generalized to the entire timber member without systematic replicate sampling. Quantitative image analysis of crack density, void area, wall fragmentation and surface texture was not performed. EDS values are field-specific and semi-quantitative rather than describing bulk composition. Although the instrument, SEM imaging conditions, EDS voltage, beam current, acquisition time and uncoated state of transverse sections are reported, several archived EDS metadata fields were unavailable, including detector geometry, dead time, map pixel dimensions and matrix-correction settings. Surface roughness and porosity can also affect interaction volume and apparent X-ray intensity. Finally, SEM–EDS does not identify crystalline phases, salt species or their concentrations. The SEM–EDS findings therefore support comparative, hypothesis-generating interpretation and not compound-level or causal attribution.

Future research should therefore combine MLC or cone-calorimeter testing with controlled moisture conditioning, larger specimen numbers, density-matched contemporary references and stratified sampling of surface and inner layers, supplemented by quantitative microscopy and phase-specific chemical methods [21,22,23]. Particularly important would be the separation of three effects that are partly superimposed in historic timber: natural ageing, biological or abiotic degradation, changes in the chemical composition of individual wood components and previous thermal exposure. Such work would allow a more quantitative model of how surface condition modifies ignition while char formation and material density control cumulative heat release.

## 6. Conclusions

The conclusions below apply to the tested oak materials and to the specified MLC conditions. They should be transferred to other historic timber elements only after verification of species, density, moisture state, surface condition and contamination profile.

Contemporary oak retained the most regular and readable SEM microstructure, with clear lumina, relatively continuous cell-wall boundaries and lower surface heterogeneity.

Aged oak preserved recognizable anatomy but exhibited increased roughness, fibrillation, local delamination, fractured wall edges and attached particles, indicating heterogeneous near-surface degradation.

Charred historic oak showed the strongest alteration, including secondary porosity, fragmentation, brittle surface morphology and partial loss of cell-wall continuity.

SEM–EDS showed that the analyzed preserved historic fields were dominated by C and O, whereas selected degraded and charred fields contained a broader group of locally distributed inorganic elements, including Na, Mg, Al, Si, P, S, Cl, K, Ca, Ti and Fe. These results demonstrate microscale elemental heterogeneity but do not identify specific salts or mineral phases.

Surface degradation, rather than age alone, explains the shorter ignition time of historic wood; roughness, thin fibrils, fractured edges, loose particles and secondary pores may act as local ignition-sensitive points.

Faster ignition did not correspond to higher total heat release or greater final mass loss. Ignition sensitivity and overall fire contribution should therefore be assessed separately.

Historic oak should be evaluated according to its actual preservation state, integrating SEM, SEM–EDS, density, moisture content and fire-test data with conservation objectives.

Overall, the study shows that the fire behaviour of historic oak is governed primarily by the condition of the surface and near-surface zone. Age alone is not sufficient to classify historic wood as less safe or more fire-prone. Roughness, secondary porosity, fragmentation and mineral enrichment may shorten ignition time, but they do not necessarily increase total heat release. Therefore, assessment of historic oak should be condition-based and should combine microscopic, chemical and fire-performance data.

## Figures and Tables

**Table 1:** Material groups used in the comparative interpretation.

Material/Variant	Origin and Condition	Density and Moisture Information	Role in Analysis
QN—contemporary oak	New oak supplied by university workshops; no visible defects.	Average density approx. 737.03 kg/m^3^; moisture 7–8%.	Reference material for MLC and SEM comparison.
QHND—historic oak, non-degraded surface	Oak from Swołowo, 1869; less damaged exposed surface within the historic set.	Reported densities approx. 580.59 and 664.10 kg/m^3^; moisture 7–8%.	Historic material with lower visible surface degradation.
QHD—historic oak, degraded surface	Oak from Swołowo, 1869; deep cracks, losses and rough damaged surface.	Reported densities approx. 590.23 and 720.07 kg/m^3^; moisture 7–8%.	Historic material used to assess the effect of strong surface degradation.

**Table 2:** Interpretation safeguards adopted to reduce overstatement of the results.

Issue Relevant to Peer Review	Safeguard Used in This Manuscript	Reason for the Safeguard
Historic material heterogeneity	The analysis distinguishes contemporary, non-degraded historic, degraded historic and charred/degraded zones.	Prevents treating historic oak as a uniform age-defined category.
Limited destructive sampling	Results are interpreted as condition-based trends rather than universal material constants.	Reflects constraints typical of heritage timber research.
SEM image selection	SEM is used for comparative mapping and mechanism building, not for quantitative stereology.	Avoids unsupported claims about absolute porosity or roughness.
SEM–EDS interpretation	Elements are discussed separately from mineral phases and salts.	EDS detects elements, not crystalline compounds.
Fire-test interpretation	TTI, HRR, THR and mass loss are interpreted as different response dimensions.	Prevents equating faster ignition with higher total fire contribution.

**Table 3:** Average MLC results with standard deviation in brackets.

Parameter	QN 35	QHND 35	QN 50	QHND 50	QHD 50
Max. HRR [kW/m^2^]	134 (8.7)	120 (13.1)	130 (3.8)	120 (1.1)	140 (3.6)
n	5	6	5	6	6
Max. ARHE [kW/m^2^]	92 (6.6)	76 (8.8)	98 (1.1)	93 (1.4)	115 (1.23)
THR [MJ/m^2^]	310 (9.8)	225 (18.3)	263 (13.1)	208 (8.2)	233 (4.1)
EHOC [MJ/kg]	77 (2.0)	76 (1.5)	76 (2.5)	76 (1.2)	79 (0.4)
Max. MLR [g/s]	0.26 (0.02)	0.20 (0.03)	1.19 (1.3)	0.25 (0.04)	0.26 (0.04)
Mass loss [%]	73 (0.6)	73 (0.05)	74 (0.2)	74 (0.1)	73 (0.4)
TTI [s]	56.67 (8.3)	50.33 (5.3)	25.33 (2.4)	18 (3.6)	5.33 (0.9)

Abbreviations: HRR—heat release rate (rate of heat release); Max. HRR—maximum heat release rate; n—number of replicates; ARHE—average rate of heat emission; Max. ARHE—maximum average rate of heat emission; THR—total heat release; EHOC—effective heat of combustion; MLR—mass loss rate; Max. MLR—maximum mass loss rate; Mass loss—percentage mass loss after the test; TTI—time to ignition.

**Table 4:** Comparative SEM-based diagnostic features of contemporary, aged and charred oak wood.

Feature	Contemporary Oak	Aged Oak	Charred Oak	Interpretative Significance
Anatomical readability	High; lumina and boundaries clearly identifiable.	Moderate; anatomy remains visible but locally obscured.	Low to moderate; fibre orientation partly preserved but strongly altered.	Lower readability indicates degradation of the surface and near-surface network.
Cell-wall continuity	Relatively continuous rims around vessels and lumina.	Locally fractured edges and discontinuous wall fragments.	Strongly discontinuous, brittle and fragmented wall remnants.	Discontinuity increases thin exposed edges that may heat rapidly.
Surface roughness	Low to moderate, mostly preparation-related.	Moderate to high, with uneven relief and fibrillation.	Very high, with jagged edges, microvoids and charred relief.	Roughness increases active surface area and promotes local heating.
Porosity	Mostly primary anatomical porosity.	Primary porosity with local secondary voids and damaged cavities.	Primary porosity overprinted by cracks and secondary voids.	Irregular porosity can alter oxygen and heat access.
Particles/deposits	Limited.	Present locally.	Common in degraded or charred regions.	Deposits reflect service history and can modify local response.

**Table 5:** SEM–EDS summary of detected elements and interpretative relevance.

Sample/Zone	Detected Elements or Reported Values	Main Interpretation	Caution for Interpretation
Aged oak, relatively preserved longitudinal areas	C = 53.79–58.55 wt.%; O = 40.64–45.04 wt.%; trace Na, Al, Si, S, Cl, K and Ca.	Predominantly organic lignocellulosic matrix with limited mineral contamination.	Trace elements should not be overinterpreted without phase identification.
Aged oak, transverse section	C = 66.88 wt.%; O = 32.16 wt.%; minor K and trace Na.	Organic character of less degraded or inner regions is largely preserved.	Values are field-specific and should not be generalized to all historic wood.
Degraded aged surface	Elevated Si, Ca, Fe and Al reported in the degraded surface area.	Local inorganic enrichment compatible with environmental particles, contact with mineral building materials or Fe-rich deposits.	EDS detects elements but does not identify specific minerals, salts or corrosion phases.
Charred historic oak surface	C, O, N, Na, Mg, Al, Si, P, S, Cl, K, Ca, Ti and Fe.	Greater local elemental diversity and chemical heterogeneity in the altered surface zone.	Possible concentration of non-volatile constituents and retained contamination cannot be separated quantitatively.
Element co-localization	Possible Na–Cl, Ca–S, Si–Al and Fe–O spatial associations.	Supports selection of individual deposits for targeted phase-specific analysis.	Co-localization is not compound identification; confirmation requires XRD, Raman, FTIR or ion chromatography.

**Table 6:** Strength-of-evidence matrix linking observations with interpretation.

Interpretative Claim	Primary Evidence	Level of Support	Reviewer-Safe Formulation
Degraded historic surfaces ignite earlier.	TTI is reduced for QHND and especially QHD at 50 kW/m^2^.	Strong within this dataset.	Surface condition is a plausible controlling factor for early ignition.
Roughness and fibrillation promote local heating.	SEM shows thin fragments, cracked edges and fibrillated walls.	Mechanistically plausible.	These features can create ignition-sensitive points.
Historic oak has lower total heat release in tested variants.	THR values are lower for QHND and QHD than for QN in comparable tests.	Strong for reported variants.	Earlier ignition did not translate into higher THR under these conditions.
Mineral enrichment occurs in degraded and charred zones.	SEM–EDS detects Na, Mg, Al, Si, P, S, Cl, K, Ca, Ti and Fe in altered zones.	Moderate; field-specific.	Degraded surfaces are chemically heterogeneous and require phase-specific confirmation.
Age alone is not an adequate risk criterion.	Contrasting behaviour of non-degraded and degraded historic surfaces.	Strong conceptual support.	Assessment should be condition-based rather than age-based.

## Data Availability

The original contributions presented in this study are included in the article. Further inquiries can be directed to the corresponding author.
